# Cerebral Amyloid Angiopathy With a Hypomanic Episode Treated With Valproic Acid

**DOI:** 10.7759/cureus.16411

**Published:** 2021-07-15

**Authors:** Naomichi Okamoto, Atsuko Ikenouchi, Issei Seki, Natsumi Hirano, Reiji Yoshimura

**Affiliations:** 1 Medical Center for Dementia, University Hospital/Psychiatry, University of Occupational and Environmental Health, Kitakyushu, JPN; 2 Psychiatry, University of Occupational and Environmental Health, Kitakyushu, JPN; 3 Radiology, University of Occupational and Environmental Health, Kitakyushu, JPN

**Keywords:** cerebral amyloid angiopathy (caa), hypomanic episode, valproic acid, mild cognitive impairment, alzheimer’s disease (ad)

## Abstract

Cerebral amyloid angiopathy (CAA) is a major cause of spontaneous intracerebral hemorrhage in the elderly. There are no reports of CAA causing mania or hypomanic episodes, and the incidence of mania or hypomanic episodes in patients with vascular dementia is generally considered to be rare. Here, we present a case of CAA with hypomania in which valproic acid (VPA) led to improvement in the symptoms of hypomania. An 80-year-old, right-handed Japanese woman with mild cognitive impairment (MCI) presented with a hypomanic episode. Her brain magnetic resonance imaging showed multiple obsolete infarcts and hemorrhages caused by CAA. We diagnosed her as suffering from a hypomanic episode in MCI associated with CAA and started VPA 400 mg/day for seven weeks. Her hypomanic episode gradually improved on VPA. This case indicates that VPA can be useful in the treatment of hypomanic episodes in MCI associated with CAA.

## Introduction

Cerebral amyloid angiopathy (CAA) is characterized by the accumulation of amyloid fibrils in the walls of small to medium-sized arterial blood vessels, central nervous system parenchyma, and leptomeningeal capillaries. CAA is a major cause of spontaneous intracerebral hemorrhage in the elderly and is an important factor in age-related cognitive decline [1]. While CAA is a frequent pathological change in Alzheimer’s disease (AD), it is sometimes treated as an independent clinical entity. The sentinel clinical presentations of CAA include cognitive impairment and dementia and transient focal neurological episodes often associated with acute convexity subarachnoid hemorrhage or cortical superficial siderosis [2]. Regarding cognitive impairment, which is a common symptom, compared to AD, patients with CAA have better memory retention, but a marked decline in executive function and processing speed, which is similar to symptoms seen in vascular dementia [3]. To date, mania or hypomanic state caused by CAA with mild cognitive impairment (MCI) has not been reported. Here, we present a case of a hypomanic episode in MCI associated with CAA. To the best of our knowledge, this is the first report to show the efficacy of valproic acid (VPA) to treat hypomanic episodes in MCI associated with CAA.

## Case presentation

**Figure 1:**
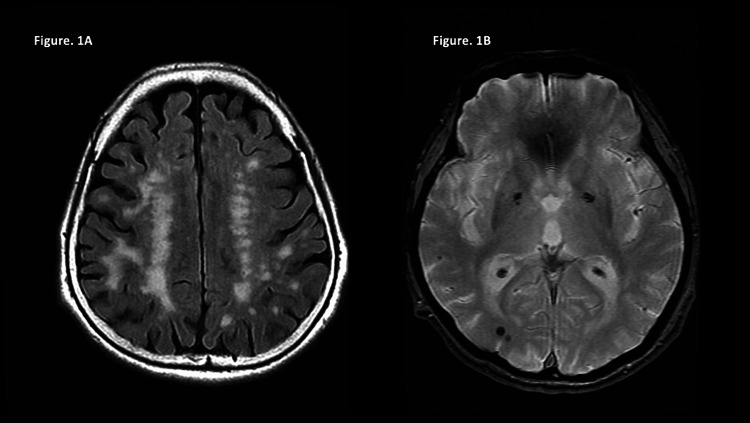
Brain MRI. (A) High signal intensity can be seen in the bilateral cerebral white matter, indicating chronic ischemic changes on T2WI/FLAIR. (B) Low signal intensity can be seen in bilateral temporal lobes, occipital lobes, right subcortical frontal lobes, and right illuminating hemisphere, indicating obsolete microbleeds and CAA on T2WI. CAA: cerebral amyloid angiopathy; T2WI: T2-weighted image; FLAIR: fluid-attenuated inversion recovery

An 80-year-old, right-handed Japanese woman made an outpatient visit to our university hospital. Her initial symptoms were poor judgment, low concentration, and personality changes, which started at the age of 79. She had a physical history of hypertension and uterine fibroids. She had no personal or family history of psychiatric diseases, such as depression or mania She was able to maintain her daily life, and her Mini-Mental State Examination-Japanese (MMSE-J) score was 29/30 points. There were no obvious neurological findings. A head computed tomography (CT) was performed which showed mild atrophy of the bilateral cerebrum, old infarcts in the left basal ganglia, and deep white matter of the left frontal lobe. As a result, dementia was ruled out and she was diagnosed with MCI. She was kept on observation without anti-dementia drugs; however, gradually, she became irritative. At the age of 80, her family noticed her hypomanic episodes such as verbosity, flight of ideas, hypertrophy of self-esteem, mood swings, irritability, excessive energy, and distraction. There was no decrease in the desire for sleep, sexual hyperactivity, or fanciful dressing. As a result, her family brought her to our hospital again. At this time, her MMSE-J score was 27/30 points, Montreal Cognitive Assessment-Japan (MoCA-J) score was 19/30 points, and Young Mania Rating Scale (YMRS) score was 25/60 points. There was no visual hallucination, muscle rigidity, parkinsonism, antisocial, and homophobic behavior. Hematological and biochemical examinations as well as thyroid function were normal. Her brain magnetic resonance imaging (MRI) showed diffuse cerebral atrophy involving the medial temporal lobe. T2-weighted image/fluid-attenuated inversion recovery (T2WI/FLAIR) showed a high signal intensity in the bilateral cerebral white matter, indicating chronic ischemic changes due to lacunar infarction (Figure 1A). In addition, T2WI showed low signal intensity in bilateral temporal lobes, occipital lobes, right subcortical frontal lobes, and right illuminating hemisphere, indicating obsolete microbleeds and CAA from the distribution (Figure 1B). The patient was diagnosed with probable CAA according to the Boston criteria [4], along with a hypomanic episode in the setting of MCI associated with CAA. We started her on VPA 400 mg/day for the hypomanic episode. Her hypomanic episode was gradually improved, and her YMRS score decreased to 8/60 points seven weeks after VPA was initiated. Her blood level of VPA was 43.1 µg/mL. Although she still has some symptoms of MCI and mild verbosity, she does not worry about her daily living and continues to visit our outpatient department.

## Discussion

The following two important findings were noted in this case: MCI from CAA can present as a hypomanic episode, and VPA can be effective in the treatment of the hypomanic episode associated with CAA. The frequency of mania in patients with vascular dementia is generally considered to be rare, for example, in a study involving 500 consecutive stroke patients, there were only two cases of mania [5]. In other studies, patients had a history of bipolar disorder [6] or were on antidepressant drugs [7]. Strong evidence shows that VPA is effective in mania of bipolar disorder [8]. On the other hand, a systematic meta-analysis found insufficient evidence to support VPA to treat dementia for cognitive, psychiatric, or disease-modifying symptoms [9]. Therefore, in this case, the symptoms were indeed related to mania rather than personality changes associated with behavioral and psychological symptoms of dementia.

CAA is caused by the accumulation of amyloid-β protein in the walls of cortical and leptomeningeal arteries [1]. Lobar microbleeds are a hallmark of the disease, which were also observed in this case [10]. Although frequently associated with AD, CAA also independently contributes to cognitive impairment [11]. The Boston criteria are non-invasive radiological criteria [4] to differentiate and diagnose CAA. We used history, MoCA-J, and MMSE-J to rule out AD in this patient. Classically, Starkstein and Robinson reported that post-stroke mania is strongly associated with both a right hemisphere lesion in a limbic-connected area and a second predisposing factor, such as genetic loading for affective disorder, pre-existing subcortical atrophy, or seizure disorder [12]. In our case, T2WI showed low single intensity right subcortical frontal lobes and right illuminating hemisphere. VPA can potentiate the inhibitory effect of γ-aminobutyric acid (GABA) transmission, possibly by enhancing GABA synthesis and blocking metabolism [13]. In this case, VPA was effective in treating hypomania due to CAA as well as bipolar disorder, suggesting that it reinforces the pathology that mania is the result of neuroexcitatory conduction due to abnormal brain function. In addition, VPA suppresses inflammatory cytokines such as interleukin-1beta and tumor necrosis factor-alpha [14] and reduces amyloid-β oligomers, leading to loss of synaptic proteins from the neuron [15]. Hence, VPA can have an additional and different pharmacological effect on amyloid to improve amyloid-β deposition.

There are several limitations to this study. CAA and AD cannot be definitively diagnosed without a pathological autopsy. In our case, T2WI showed low signal intensity in the right illuminating hemisphere as well as bilateral temporal lobes.

## Conclusions

We presented the case of an 80-year-old, right-handed Japanese woman with MCI caused by CAA. She presented with a hypomanic episode, and VPA was effective in the treatment of the disease. To the best of our knowledge, this is the first report of MCI from CAA that presented with a hypomanic episode, even though there was no history of bipolar disorder or antidepressant drug use. Second, VPA can be useful in the treatment of hypomanic episodes. Further studies should be conducted to confirm the effectiveness of VPA.
